# Perinatal Mental Health in Women with Polycystic Ovary Syndrome: A Cross-Sectional Analysis of an Australian Population-Based Cohort

**DOI:** 10.3390/jcm8122070

**Published:** 2019-11-25

**Authors:** Chau Thien Tay, Helena J. Teede, Jacqueline A. Boyle, Jayashri Kulkarni, Deborah Loxton, Anju E. Joham

**Affiliations:** 1 Monash Centre for Health Research and Implementation, School of Public Health and Preventive Medicine, Monash University, Clayton VIC 3168, Australia or jillian.cttay@gmail.com (C.T.T.); helena.teede@monash.edu (H.J.T.); jacqueline.boyle@monash.edu (J.A.B.); 2 Department of Diabetes and Vascular Medicine, Monash Health, Clayton VIC 3168, Australia; 3 The Monash Alfred Psychiatry Research Centre, Monash University and the Alfred Hospital, Melbourne VIC 3004, Australia; jayashri.kulkarni@monash.edu; 4 Research Centre for Generational Health and Ageing, School of Medicine and Public Health, University of Newcastle, Callaghan NSW 2308, Australia; Deborah.loxton@newcastle.edu.au

**Keywords:** polycystic ovary syndrome, perinatal mental health, depression, anxiety

## Abstract

Women with polycystic ovary syndrome (PCOS) have many risk factors associated with perinatal mental disorders, but research in this area is scarce. This study aims to compare the prevalence of common perinatal mental disorders in women with and without PCOS, and examine the relationship between PCOS and common perinatal mental disorders. We performed a cross-sectional study on self-reported data of 5239 women born between 1973 to 1978 in the Australian Longitudinal Study on Women’s Health. Compared with women not reporting PCOS, women reporting PCOS had higher prevalence of antenatal depression (8.9% vs. 4.4%, *p* < 0.001), antenatal anxiety (11.7% vs. 5.6%, *p* < 0.001), postnatal depression (26.8% vs. 18.6%, *p* < 0.001) and postnatal anxiety (18.4% vs. 12.0%, *p* < 0.001). PCOS was positively associated with antenatal depression and/or anxiety (adjusted odds ratio 1.8, 95% confidence interval 1.2–2.6) but not postnatal depression and/or anxiety after controlling for sociodemographic and lifestyle factors, reproductive history, obstetric complications and pre-existing depression and anxiety. General perinatal guidelines currently do not recognize PCOS as a risk factor and the international evidence based PCOS guideline noted inadequate evidence in this area. This paper addresses the gap in literature and highlights the need to screen for common perinatal mental disorders in women with PCOS.

## 1. Introduction

Perinatal mental disorders are among the most prevalent morbidities of pregnancy and the post-partum period and occur in one in every five women [1,2]. While postnatal depression is the most studied perinatal mental disorder, other common perinatal mental disorders include antenatal depression, antenatal anxiety and postnatal anxiety [3]. Perinatal mental disorders impact adversely on the well-being of both women and their offspring. Short-term effects include increased pre-term birth [4,5,6,7], low birth weight [4,5,6,7], pre-eclampsia [8,9] and poor mother-infant interactions during the immediate postnatal period [6,10,11]. Emerging literature from longitudinal studies also shows that perinatal mental disorders have deleterious effects on longer term outcomes in the offspring including poorer academic achievement and increased risk for psychopathology in their adolescent years [12,13,14,15,16,17].

Identifying women at risk of perinatal mental disorders is important as screening and subsequent interventions have been shown to be effective in preventing the development of perinatal mental disorders and improving symptom severity in the women [18,19,20]. Although lacking long-term data, treatment of these disorders also improves the offspring’s psychomotor and behavioural development [21,22,23]. Evidence from systematic reviews and meta-analyses have consistently shown that pre-existing mental health disorder is a major risk factor for poor perinatal mental health [1,24,25,26,27]. Other potential relevant risk factors include sociodemographic factors (abuse, stable relationship, financial difficulties etc.), obstetric complications and the use of reproductive therapies [28,29,30,31].

Polycystic ovary syndrome (PCOS) is a complex endocrinopathy characterized by oligomenorrhoea, hyperandrogenism and polycystic ovary morphology on ultrasound that affects 8–13% of young women [32,33]. It is associated with a wide range of reproductive (menstrual irregularity, anovulation), endocrine (impaired glucose tolerance, type 2 diabetes) and metabolic (metabolic syndrome, fatty liver, increased cardiovascular risk factors) complications [34,35,36,37,38]. Mental health disorders such as depression, anxiety and eating disorders are prevalent in women with PCOS and psychological screening is advocated by the latest International evidence-based guideline for the assessment and management of PCOS [39,40]. PCOS is also the most common cause of anovulatory infertility with up to 70% of women with PCOS experiencing infertility and nearly 90% of them seeking fertility treatment [41]. Furthermore, women with PCOS have high risks for obstetric complications such as gestational diabetes, hypertensive disorders in pregnancy, pre-eclampsia, pre-term birth and infants requiring admission to a neonatal intensive care unit [42].

Women with PCOS have many risk factors for perinatal mental disorders but research regarding perinatal mental health in this population is lacking and recognized as a major evidence gap in the Centre of Research Excellence (CRE) in PCOS led International PCOS guideline. To date, only one other study examined postnatal depression in women with PCOS [43]. We hypothesized that women with PCOS have higher prevalence of perinatal mental disorders. We aim to investigate the prevalence of common perinatal mental disorders (specifically antenatal depression, antenatal anxiety, postnatal depression and postnatal anxiety) in women with and without PCOS in an unselected and representative community recruited cohort. We also aim to explore the relationship between PCOS status and perinatal mental disorders taking into account the women’s reproductive history, obstetric complications and pre-existing depression and anxiety.

## 2. Methods

### 2.1. Study Population and Methods

The Australian Longitudinal Study on Women’s Health (ALSWH) is a longitudinal population-based survey started in 1996 which assesses women’s physical and mental health, sociodemographic factors, lifestyle and use of health services to inform health policy decision making [44]. The original study involves women from three age cohorts who were then aged 18–23, born 1973–1978; aged 45–50, born 1946–1951 and aged 70–75, born 1921–1926. Women were randomly selected and invited to participate via mail from the Medicare database, a national health insurance scheme which includes all Australian citizens and permanent residents. Intentional oversampling from rural and remote areas were performed to study health experiences of women from non-metropolitan areas [44]. In 2012 and 2013, a fourth cohort of women aged 18–23, born 1989–1995, was added to the study [45]. Ethical approval was obtained from the Human Research Ethics Committees from the University of Queensland, the University of Newcastle, Medicare and the Department of Health. Further details and full survey content of the ALSWH are available at www.alswh.org.au.

For the purpose of this study, we utilized data of the cohort of women born between 1973–1978. We performed a cross-sectional analysis of the data from Survey 7 collected in 2015, but data on PCOS status and pre-existing mental disorders were obtained from Surveys 4 to 7 (collected in 2006, 2009, 2012 and 2015). We excluded from our analysis women with missing information about PCOS status, perinatal mental health history, parity history and women who have never given birth.

### 2.2. Measures

#### 2.2.1. Outcome Variables

*Perinatal mental disorder.* Women who have given birth to a child before were surveyed for perinatal mental health. The specific perinatal mental disorders assessed in the survey were antenatal depression, antenatal anxiety, postnatal depression and postnatal anxiety by using the question, “were you diagnosed with or treated for…”. Specific definitions of the antenatal and postnatal period were not provided in the survey. Women were considered to have a history of perinatal mental disorder if they responded “yes” to the conditions for any births they had experienced.

#### 2.2.2. Explanatory Variables

*Polycystic ovary syndrome (PCOS).* Women were asked, “in the last 3 years, have you been diagnosed with or treated for PCOS?” from Surveys 4 to 7. Women were considered to have a history of PCOS if they responded “yes” to the question. This self-reported status of PCOS has previously been validated against menstrual irregularity, a key symptom of PCOS [46].

*Body mass index (BMI).* Self-reported height and weight were collected in Survey 7 and BMI was calculated using the formula, weight in kilograms divided by the square of height in meters. We categorized BMI in accordance to the World Health Organization’s criteria: BMI < 18.5 is classified as underweight, BMI 18.5–24.9 is healthy, BMI 25.0–29.9 is overweight and ≥30 is obese [47]. Self-reported BMI has been previously validated with direct measurements in the ALSWH [48].

*Reproductive history.* Women who reported having been pregnant before were asked about the number of times they had a miscarriage and/or termination of pregnancy. These were treated as binary variables in our analysis. Infertility was defined as trying unsuccessfully for 12 months or more to become pregnant and assessed by the question, “have you or your partner (current or previous) had ever had problems with fertility?”. Women who chose the responses “yes, but have not sought help/treatment” or “yes, and have sought help/treatment” were classified as having experienced infertility. For fertility treatment, women who responded “yes” to “I am using/have used In Vitro Fertilization (IVF)” or “I am using/have used fertility hormones (e.g., Clomid)” were classified as having received fertility treatment.

*Obstetrics complications.* Women who have given birth to a child were asked about pregnancy complications including gestational diabetes, hypertensive disorders in pregnancy and pre-term labour (defined as labour before 36 weeks gestation), and birth complications including induction of labour, instrumental delivery, caesarian section, low birth weight infant (defined as <2.5 kg), high birth weight infant (defined as >4 kg), infant requiring admission to Special Care Nursery/Neonatal Intensive Care Unit, and stillbirth (defined as death of a fetus in utero of at least 20 weeks gestation or at least 400 g birth weight). Women were considered to have a history of the specified obstetric complication if they responded “yes” to the conditions for any births they had experienced.

*Social support.* The 19 item Medical Outcomes Study (MOS) Social Support Survey [49] was embedded in Survey 7. This validated tool uses 5 point Likert scale to assess tangible support, affectionate support, positive social interaction and emotional/informational support. The responses range from “none of the time” to “all of the time”. Total score ranges from 1 to 5 and we categorized mean scores of ≤3 as “low level of support”, >3 to ≤4 as “moderate level of support” and >4 as “high level of support”.

Other explanatory variables included age, education level, occupation, marital status, area of residence, smoking and alcohol drinking pattern.

#### 2.2.3. Statistical Analysis

Summary statistics were presented using means with standard deviation or median with interquartile range for continuous data and percentages for categorical data. Differences in variables between women reporting PCOS and women not reporting PCOS were tested using *t*-test, χ2 test or Mann–Whitney U test as appropriate. Logistic regression analyses were performed to examine the relationships between PCOS with reproductive history, obstetric complications and perinatal mental disorders. Multivariable logistic regression analyses for individual common perinatal mental disorders were not performed due to small number of events in individual groups. We performed separate adjusted analysis to examine the relationship between PCOS with antenatal depression and/or anxiety and postnatal depression and/or anxiety. Covariates included in the regression analysis included factors known or suspected to have an effect on perinatal mental disorders, and/or exhibiting *p* < 0.1 on simple regression analysis. Area of residence was also included in the analysis to adjust for the deliberate oversampling of women in rural and remote areas. Reproductive history, obstetric complications and pre-existing mental disorders were treated as confounders rather than mediators in the adjusted models. Final included covariates included age, BMI education, occupation, marital status, smoking and alcohol habits, social support, area of residence, reproductive history, obstetric complications and pre-existing anxiety and depression. Potential interactions between PCOS with reproductive history, obstetric complication and pre-existing mental disorders were examined by including their interaction terms in the multivariable logistic regression models. Participants with missing covariates were not included in the multivariable regression analyses. All *p* values were calculated using two-tailed test where *p* < 0.05 is considered statistically significant. All analyses were performed using Stata version 15.

## 3. Results

### 3.1. Study Population and Their Characteristics

**Figure 1:**
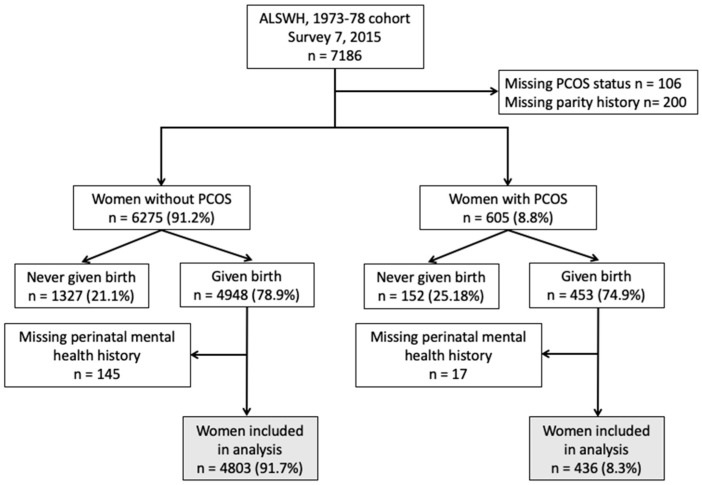
CONSORT diagram of women included in the final analysis.

Overall, 7186 women between age 37 and 42 years participated in Survey 7 with 106 women and 200 women excluded due to missing data on PCOS status and parity respectively. Of the remaining 6880 women, a further 1479 women were excluded from our study due to never having given birth to a child. Among them, 162 women had incomplete histories on perinatal mental health. Finally, a total of 5239 women were included in our analysis where 436 women reported PCOS (8.3%, 95% confidence interval (CI) 7.6–9.1%) (Figure 1). All the participants excluded due to missing information had similar age and BMI to participants included in the study, but they were less well educated (results not shown).

Table 1 presents the characteristics of the study population. The mean age of the overall population was 39.7 (±1.5) years with the majority (88.1%) of the women either married or in a de facto relationship. Both groups had similar education levels, occupation, marital status, social support, smoking history, alcohol drinking pattern and area of residence. Women reporting PCOS had higher mean BMI than women not reporting PCOS (29.3 ± 7.7 vs. 26.4 ± 6.0 kg/m^2^, *p* < 0.001). The prevalence of pre-existing depression (39.5% vs. 25.5%, *p* < 0.001) and anxiety (27.5% vs. 17.2%, *p* < 0.001) were also more common in women reporting PCOS compared with women not reporting PCOS.

### 3.2. Reproductive History and Their Relationship with PCOS

The number of live births per woman across the whole study population ranged between 0 and nine. Although there was no difference in the median number of live births (median = 2) between the groups, two-thirds (60.2%) of women reporting PCOS reported having experienced infertility at some time in their life and their odds for infertility was six times higher than women not reporting PCOS (odds ratio (OR): 6.3, 95% CI 5.1–7.8) (Table 2). Significantly higher proportions of women reporting PCOS also reported having used hormonal (27.8% vs. 5.5%, *p* < 0.001) or IVF (18.6% vs. 6.6%, *p* < 0.001) fertility treatment with associated increased odds of 6.7 (95% CI 5.2–8.5) and 3.3 (95% CI 2.5–4.3) respectively (Table 2). A history of miscarriage was more common in the PCOS group (43.7% vs. 35.9%, *p* = 0.001; OR 1.4, 95% CI 1.1–1.7), but the rate of termination of pregnancy did not differ significantly between groups (Table 2).

### 3.3. Obstetric Complications and Their Relationship with PCOS

Significantly higher prevalence and odds ratios for gestational diabetes (16.3% vs. 9.0%, *p* < 0.001; OR: 2.0, 95% CI 1.5–2.6), hypertensive disorders in pregnancy (19.5% vs. 13.8%, *p* = 0.001; OR: 1.5, 95% CI 1.2–1.9), pre-term labour (18.7% vs. 10.0%, *p* < 0.001; OR: 2.1, 95% CI 1.6–2.7), caesarean section (36.1% vs. 30.1%, *p* = 0.009; OR 1.3, 95% CI 1.1–1.6) and low birth weight < 2.5 kg (15.4% vs. 9.0%, *p* < 0.001; OR: 1.8, 95% CI 1.4–2.4) were observed in women reporting PCOS compared with women not reporting PCOS (Table 2). More women reporting PCOS also had given birth to an infant requiring admission under special care nursery or neonatal intensive care unit (27.1% vs. 18.1%, *p* < 0.001), roughly twice the odds when compared with women not reporting PCOS (OR: 1.7, 95% CI 1.4–2.2). There was no observed difference in rates of induction of labour, instrumental delivery, high birth weight infants or stillbirths between the groups (Table 2).

### 3.4. Common Perinatal Mental Disorders and Their Relationship with PCOS

24.6% women reported a history of a common perinatal mental disorder and this is observed more commonly in women reporting PCOS than women not reporting PCOS (33.5% vs. 23.8%, *p* < 0.001) (Table 3). Specifically, the prevalence was 4.7% for antenatal depression (8.9% vs. 4.4%, *p* < 0.001), 6.1% for antenatal anxiety (11.7% vs. 5.6%, *p* < 0.001), 19.3% for postnatal depression (26.8% vs. 18.6%, *p* < 0.001) and 12.6% for postnatal anxiety (18.4% vs. 12.0%, *p* < 0.001) (Table 3). While postnatal mental disorders were more prevalent, the odds were higher in women reporting PCOS for antenatal mental disorders (OR 2.2, 95% CI 1.6–2.9) than postnatal mental disorders (OR 1.6, 95% CI 1.3–2.0) in crude analysis (Table 3).

Pre-existing depression and anxiety, low social support and being in a married or de facto relationship were positively associated with both antenatal and postnatal depression and/or anxiety (Table 4). Factors associated with antenatal depression and/or anxiety were self-reported PCOS (adjusted OR 1.8, 95%CI 1.2–2.6), overweight BMI (adjusted OR 1.3, 95% CI 1.0–1.8), unemployment (adjusted OR 1.5, 95% CI 1.1–2.1), active smoking (adjusted OR 1.8, 95% CI 1.2–2.6) and a history of stillbirth (adjusted OR 2.1, 95% CI 1.1–3.9) (Table 4). Unique to postnatal depression and/or anxiety, IVF fertility treatment was associated with reduced adjusted odds of 0.6 (95% CI 0.4–1.0).

Significant interaction was detected between PCOS and a history of IVF for postnatal but not antenatal depression and/or anxiety. In women with a history of IVF, PCOS was not associated with postnatal depression and/or anxiety. However, women reporting PCOS without a history of IVF had increased odds for postnatal depression and/or anxiety (adjusted OR 1.4, 95% CI 1.1–2.0). A positive history of stillbirth also interacted with PCOS for antenatal but not postnatal depression and/or anxiety. Women reporting PCOS without a history of stillbirth had increased adjusted odds of 2.0 (95% CI 1.4–2.9) for antenatal depression and/or anxiety. In contrast, in women with a history of stillbirth, no significant relationship was observed between PCOS and antenatal depression and/or anxiety.

## 4. Discussion

This current study is a large population-based observational study which examines self-reported data on common perinatal mental disorders in women with and without self-reported PCOS in a representative Australian cohort aged 37 to 42 years. To our knowledge, we report for the first time that women with PCOS have significantly higher prevalence of common perinatal mental disorders when compared with women without PCOS. After adjusting for sociodemographic factors, lifestyle factors, reproductive history, obstetric complications and pre-existing depression and anxiety, PCOS was positively associated with antenatal but not postnatal anxiety and/or depression. However, in women without a history of IVF, PCOS was associated with increased odds for postnatal anxiety and/or depression.

In our study population, there were no observed sociodemographic differences between women with and without PCOS, but PCOS was associated with more risk factors for perinatal mental disorders. We observed high prevalence of pre-existing depression and anxiety in women reporting PCOS compared with women not reporting PCOS and this supports mental health disorders as an integral component of PCOS [40,50]. We also observed that infertility, fertility treatment and miscarriage were more common in women reporting PCOS. In line with results from previous systematic reviews and meta-analyses, we find that PCOS was positively associated with adverse obstetric complications including gestational diabetes, hypertensive disorders in pregnancy, pre-term labour (less than 36 weeks gestation), requiring caesarean section, having an infant with low birth weight or having an infant requiring admission to a special care nursery or neonatal intensive care unit [42].

Perinatal mental health in women with PCOS is not yet widely researched. To the best of our knowledge, only one other study has been published in this area [43]. In this cross-sectional analysis of 52 women with and 514 women without PCOS, March et al. reported that postnatal depression was more common in women with PCOS (36.5% vs. 26.7%) but their results did not reach statistical significance [43]. Other common perinatal mental disorders were not investigated in their study [43]. Our study has a greater number of participants and enhanced statistical power. In addition, we investigated other common perinatal mental disorders, including women’s antenatal mental health.

Overall, we find that the prevalence of common perinatal mental disorders across the whole study population was 24.6%, similar to the previous published results in the general population [1,2]. However, we report that the prevalence of common antenatal and postnatal mental disorders was significantly higher in women with PCOS than women without PCOS. We also report the novel finding that PCOS was significantly associated with increased odds for antenatal depression and/or anxiety after adjusting for relevant factors. This finding may be due to women with PCOS having greater stress in their pregnancy due to increased risk of obstetric complications and the need for fertility treatment. For postnatal depression and/or anxiety, the positive association with PCOS was significant only in those without a history of IVF. This result is surprising given that March et al. reported that exposure to assisted reproductive treatment (ART) increased postnatal depression in women with PCOS. One potential reason for this discrepancy could be the definition of ART applied by March et al. where ovulation induction drugs, IVF and other procedures were included in the analysis.

Our study reports a significant negative association between a history of IVF treatment and postnatal depression and/or anxiety (adjusted OR 0.6, 95% CI 0.4–0.9). Previous studies examining the use of ART with perinatal mental disorders have shown conflicting results with the majority of studies reporting no association [28,30,31,51,52,53,54,55,56]. Some studies suggest that women with multiple versus singleton pregnancies were affected differently by ART (IVF or intracytoplasmic sperm injection (ICSI)), whereby women with multiple pregnancies experienced greater psychological distress [57,58,59]. One prospective cohort study reported that IVF was associated increased antenatal anxiety symptoms compared to women who conceived naturally, but postnatal anxiety or depression symptoms were similar [56]. Our study results are similar to a Finnish case-control study involving 746 couples which demonstrated less depressive and anxiety symptoms in women receiving IVF or ICSI compared with women who conceived naturally during the antenatal period to one year post-partum [53]. However, the results of our study need to be interpreted with caution as our study was unable to differentiate between multiple versus singleton pregnancy in women receiving IVF. As IVF is mainly a private health insurance funded service in Australia, it is also possible that these women in our study may be of higher socioeconomic status, which is protective for perinatal mental disorders [25,27].

An unexpected observation in our study is the positive association of common perinatal mental disorders and being married or in a de facto relationship, given that previous studies reported being a single parent is more likely to be associated with poor perinatal mental health [1,25]. However, our study only examined the marital status of the women when the survey was undertaken and did not take into account the relationship status of the women for each previous pregnancy, which may over-estimate our observed association. We also did not assess the quality of the women’s relationship with their partners in our study. Given that a poor relationship with partner is known to be a risk factor for poor perinatal mental health [1,25], this may be another reason explaining our findings.

The strengths of our study include using an unselected, population-based cohort which is broadly representative of the Australian population [45], thereby making the results of our study more generalizable. Uncommon associations were also able to be studied given the large sample size we had which improves the statistical power of our results. Most importantly, we were able to examine the relationship between PCOS and common perinatal mental disorders by taking into account significant risk factors including BMI, social support, reproductive history, obstetric complications and pre-existing depression and anxiety in addition to sociodemographic and lifestyle factors. While the study design was limited by using self-reported data, self-reported BMI and PCOS status had been shown to have good validity in previously published ALSWH studies where PCOS status had been validated against menstrual irregularly and self-reported BMI validated against measured BMI [41,46,48]. A recent meta-analysis also found that women with self-reported PCOS had the same genetic profile as women with diagnosed PCOS which further supports the use of self-reported PCOS status [60]. One other important limitation is that all explanatory and outcome variables are treated as binary variables in our study. Women’s situation changes over time and their risk factors for perinatal mental disorders may be different for each pregnancy. Our study is only exploratory in nature and we are unable to examine further the dynamic relationship of risk factors with each pregnancy. Lastly, we were unable to make any inferences regarding causal effects due to the cross-sectional nature of the study. These factors need to be taken into account when interpreting our results.

## 5. Conclusions

In conclusion, our study addresses the gaps in current literature and provided novel information confirming that women with PCOS have high prevalence of poor perinatal mental health. In particular, women with PCOS are more predisposed to antenatal mental health than postnatal mental health concerns overall but women with PCOS without a history of IVF are susceptible to postnatal mental health disorders. PCOS is not yet recognized as a risk factor for perinatal mental disorders in general perinatal clinical guidelines [19,61,62,63] and the recently published international evidence-based guideline for PCOS [39] did not address perinatal mental health due to the paucity of evidence in this area. Our findings are clinically important and significantly advances knowledge in this field by reporting women with PCOS are vulnerable to perinatal mental disorders. While our study is conducted in a large population representative of Australian women, this research should be extended to other populations in future.

## Figures and Tables

**Table 1:** Characteristics of study population.

	Overall	Non-PCOS	PCOS	*p*-Value
	*n* = 5239	*n* = 4803 (91.7%)	*n* = 436 (8.3%)	
Age (mean ± SD)	39.7 ± 1.5	39.7 ± 1.5	39.6 ± 1.4	0.058
BMI (mean ± SD)	26.7 ± 6.3	26.4 ± 6.0	29.3 ± 7.7	<0.001
BMI category n (%)				<0.001
Underweight	95 (1.8)	87 (1.8)	8 (1.9)	
Healthy	2389 (46.4)	2255 (47.8)	134 (31.2)	
Overweight	1428 (27.7)	1308 (27.7)	120 (27.9)	
Obese	1241 (24.1)	1073 (22.7)	168 (39.1)	
*Missing*	*86 (1.6)*	*80 (1.7)*	*6 (1.4)*	
Education level n (%)				0.457
Year 12 or less	837 (16.2)	768 (16.3)	69 (16.1)	
Trade/apprenticeship	122 (2.4)	114 (2.4)	8 (1.9)	
Certificate/diploma	1300 (25.2)	1179 (25.0)	121 (28.2)	
University or higher	1895 (56.2)	2664 (56.4)	231 (53.9)	
*Missing*	*85 (1.6)*	*78 (1.6)*	*7 (1.6)*	
Occupation n (%)				0.013
Manager/administrator	2786 (53.9)	2554 (53.9)	232 (53.6)	
Tradesperson/clerical	1261 (24.4)	1171 (24.7)	90 (20.8)	
Transport/production/labor	128 (2.5)	122 (2.6)	6 (1.4)	
No paid job	998 (19.3)	893 (18.8)	105 (24.3)	
*Missing*	*66 (1.3)*	*63 (1.3)*	*3 (0.7)*	
Marital status n (%)				0.368
Never married	165 (3.2)	147 (3.1)	18 (4.2)	
Married/de facto	4554 (88.1)	4183 (88.3)	371 (86.3)	
Separated/divorced/widowed	450 (8.7)	409 (8.6)	41 (9.5)	
*Missing*	*70 (1.3)*	*64 (1.3)*	*6 (1.4)*	
Smoking status n (%)				0.857
Never	3210 (61.4)	2940 (61.4)	270 (62.1)	
Ex-smoker	1521 (29.1)	1399 (29.2)	122 (28.1)	
Active smoker	494 (9.5)	451 (9.4)	43 (9.9)	
*Missing*	*14 (0.3)*	*13 (0.3)*	*1 (0.2)*	
Alcohol drinking pattern n (%)				0.069
Non-drinker	550 (10.5)	494 (10.3)	56 (12.9)	
Low risk drinker	4348 (83.2)	3988 (83.2)	360 (82.8)	
High risk drinker	330 (6.3)	311 (6.5)	19 (4.4)	
*Missing*	*11 (0.2)*	*10 (0.2)*	*1 (0.2)*	
MOS social support scale n (%)				0.429
High level	3048 (59.7)	2801 (59.9)	247 (57.9)	
Moderate level	1450 (28.4)	1329 (28.4)	121 (28.3)	
Low level	608 (11.9)	549 (11.7)	59 (13.8)	
*Missing*	*133 (2.5)*	*124 (2.6)*	*9 (2.1)*	
Area of residence n (%)				0.062
Metropolitan	2919 (56.9)	2653 (56.5)	266 (62.3)	
Regional	2085 (40.6)	1932 (41.1)	153 (35.8)	
Rural	123 (2.4)	115 (2.5)	8 (1.9)	
*Missing*	*112 (2.1)*	*103 (2.1)*	*9 (2.1)*	
Depression	1396 (26.7)	1224 (25.5)	172 (39.5)	<0.001
*Missing*	*0*	*0*	*0*	
Anxiety	945 (18.0)	825 (17.2)	120 (27.5)	<0.001
*Missing*	*0*	*0*	*0*	

SD: standard deviation; PCOS: polycystic ovary syndrome; BMI: body mass index; MOS: Medical Outcome Study.

**Table 2:** Reproductive history, obstetric complications and their unadjusted relationship with PCOS.

	Overall	Non-PCOS	PCOS	OR	95% CI
	n (%)	n (%)	n (%)		
Reproductive history n (%)					
Number of live births median (IQR)	2 (2.3)	2 (2.3)	2 (2.3)	-	-
Infertility	1187 (22.7)	925 (19.3)	262 (60.2)	6.3	5.1–7.8
Fertility treatment—hormones	383 (7.3)	262 (5.5)	121 (27.8)	6.7	5.2–8.5
Fertility treatment—IVF	395 (7.6)	314 (6.6)	81 (18.6)	3.3	2.5–4.3
Miscarriage	1903 (36.5)	1713 (35.9)	190 (43.7)	1.4	1.1–1.7
Termination of pregnancy	1029 (19.8)	958 (20.1)	71 (16.4)	0.8	0.6–1.0
Pregnancy complications n (%)					
Gestational diabetes	501 (9.6)	430 (9.0)	71 (16.3)	2	1.5–2.6
Hypertensive disorders in pregnancy	746 (14.3)	661 (13.8)	85 (19.5)	1.5	1.2–1.9
Preterm birth (<36 weeks)	555 (10.7)	474 (10.0)	81 (18.7)	2.1	1.6–2.7
Birth complications n (%)					
Induction of labour	2669 (51.5)	2439 (51.2)	230 (53.7)	1.1	0.9–1.3
Instrumental delivery	1505 (29.1)	1386 (29.2)	119 (27.8)	0.9	0.7–1.2
Caesarean section	1584 (30.6)	1427 (30.1)	157 (36.1)	1.3	1.1–1.6
Low birth weight infant (<2.5 kg)	494 (9.6)	428 (9.0)	66 (15.4)	1.8	1.4–2.4
High birth weight infant (>4 kg)	1229 (23.8)	1129 (23.8)	100 (23.4)	1	0.8–1.2
Baby requiring SCN/NICU	980 (18.9)	860 (18.1)	120 (27.1)	1.7	1.4–2.2
Stillbirths	119 (2.6)	107 (2.6)	12 (3.2)	1.2	0.7–2.3

OR: odds ratio; CI: confidence interval; IQR: interquartile range; PCOS: polycystic ovary syndrome; IVF: in vitro fertilization; SCN: special care nursery; NICU: neonatal intensive care unit.

**Table 3:** Prevalence of common perinatal mental disorders and their unadjusted relationship with PCOS.

	Overall	Non-PCOS	PCOS	Crude OR	95% CI
	n (%)	n (%)	n (%)		
Antenatal depression	248 (4.7)	209 (4.4)	39 (8.9)	2.2	1.5–3.1
Antenatal anxiety	321 (6.1)	270 (5.6)	51 (11.7)	2.2	1.6–3.1
Antenatal depression and/or anxiety	418 (8.0)	354 (7.4)	64 (14.7)	2.2	1.6–2.9
Combined antenatal depression and anxiety	151 (2.9)	125 (2.6)	26 (6.0)	2.4	1.5–3.7
Postnatal depression	1011 (19.3)	894 (18.6)	117 (26.8)	1.6	1.3–2.0
Postnatal anxiety	658 (12.6)	578 (12.0)	80 (18.4)	1.6	1.3–2.1
Postnatal depression and/or anxiety	1214 (23.2)	1077 (22.4)	137 (31.4)	1.6	1.3–2.0
Combined postnatal depression and anxiety	455 (8.7)	395 (8.2)	60 (13.8)	1.8	1.3–2.4
Any common perinatal mental disorder	1291 (24.6)	1145 (23.8)	146 (33.5)	1.6	1.3–2.0

R: odds ratio; CI: confidence interval; PCOS: polycystic ovary syndrome.

**Table 4:** Factors associated with common perinatal mental disorders.

	Antenatal Depression and/or Anxiety		Postnatal Depression and/or Anxiety	
	Adjusted OR (95% CI)	*p*	Adjusted OR (95% CI)	*p*
PCOS	1.8 (1.2–2.6)	0.002	1.2 (0.9–1.7)	0.128
Age	0.9 (0.9–1.0)	0.142	1.0 (0.9–1.0)	0.402
BMI category				
Underweight	0.8 (0.3–2.8)	0.787	0.7 (0.3–1.4)	0.311
Healthy	1		1	
Overweight	1.3 (1.0–1.8)	0.049	1.1 (0.9–1.3)	0.395
Obese	1.3 (1.0–1.8)	0.07	1.1 (0.9–1.3)	0.561
Education level				
Year 12 or less	1		1	
Trade/apprenticeship	0.9 (0.4–2.1)	0.762	0.8 (0.4–1.4)	0.4
Certificate/diploma	1.0 (0.7–1.5)	0.889	1.0 (0.8–1.3)	0.746
University or higher	1.2 (0.8–1.8)	0.29	1.0 (0.7–1.3)	0.853
Occupation				
Manager/administrator	1		1	
Tradesperson/clerical	1.1 (0.7–1.5)	0.736	1.1 (0.8–1.3)	0.657
Transport/production/labor	1.4 (0.7–3.0)	0.372	1.0 (0.6–1.8)	0.923
No paid job	1.5 (1.1–2.1)	0.011	1.2 (1.0–1.5)	0.066
Marital status				
Never married	1		1	
Married/de facto	2.8 (1.2–6.4)	0.014	2.3 (1.4–3.9)	0.001
Separated/divorced/widowed	1.9 (0.8–4.5)	0.172	1.6 (0.9–2.9)	0.08
Smoking status n				
Never	1		1	
Ex-smoker	1.1 (0.9–1.5)	0.345	1.1 (0.9–1.4)	0.212
Active smoker	1.8 (1.2–2.6)	0.003	1.2 (0.9–1.6)	0.167
Alcohol drinking pattern				
Non-drinker	1		1	
Low risk drinker	0.7 (0.5–1.1)	0.109	0.9 (0.7–1.1)	0.342
High risk drinker	0.8 (0.4–1.3)	0.354	0.9 (0.6–1.4)	0.733
MOS social support scale				
High level	1		1	
Moderate level	1.3 (1.0–1.7)	0.057	1.3 (1.0–1.5)	0.013
Low level	1.4 (1.0–2.0)	0.052	1.3 (1.0–1.7)	0.033
Area of residence				
Metropolitan	1		1	
Regional	0.8 (0.6–1.0)	0.06	1.0 (0.8–1.1)	0.681
Rural	0.8 (0.4–1.7)	0.538	0.8 (0.5–1.4)	0.426
Reproductive history				
Infertility	1.0 (0.7–1.3)	0.783	1.0 (0.8–1.2)	0.781
Fertility treatment—hormones	0.9 (0.5–1.5)	0.603	1.2 (0.8–1.8)	0.307
Fertility treatment—IVF	0.9 (0.5–1.6)	0.803	0.6 (0.4–1.0)	0.028
Miscarriage	1.0 (0.8–1.3)	0.946	1.1 (0.9–1.2)	0.543
Termination of pregnancy	1.0 (0.8–1.4)	0.839	1.0 (0.8–1.2)	0.862
Pregnancy complications				
Gestational diabetes	1.1 (0.8–1.7)	0.479	1.1 (0.9–1.5)	0.31
Hypertensive disorders in pregnancy	1.3 (0.4–1.1)	0.057	1.1 (0.9–1.4)	0.363
Preterm birth (<36 weeks)	0.7 (0.4–1.1)	0.122	1.0 (0.7–1.4)	0.953
Birth complications				
Induction of labour	1.1 (0.9–1.4)	0.376	1.1 (0.9–1.3)	0.353
Instrumental delivery	1.2 (0.9–1.6)	0.192	1.0 (0.9–1.2)	0.721
Caesarean section	1.1 (0.8–1.4)	0.656	1.0 (0.8–1.2)	0.881
Low birth weight infant (<2.5 kg)	1.2 (0.7–1.9)	0.522	0.9 (0.6–1.2)	0.45
High birth weight infant (>4 kg)	1.2 (0.9–1.6)	0.135	1.0 (0.8–1.2)	1
Baby requiring SCN/NICU	1.3 (1.0–1.8)	0.08	1.2 (0.9–1.4)	0.17
Stillbirths	2.1 (1.1–3.9)	0.024	1.1 (0.7–1.9)	0.674
Pre-existing mental disorders				
Depression	3.3 (2.5–4.3)	<0.001	5.3 (4.4–6.4)	<0.001
Anxiety	2.2 (1.7–2.9)	<0.001	2.0 (1.6–2.4)	<0.001

OR: odds ratio; CI: confidence interval; *p: p*-value; PCOS: polycystic ovary syndrome; BMI: body mass index; MOS: medical outcome study; IVF: in vitro fertilization; SCN: special care nursery; NICU: neonatal intensive care unit.
